# The Present Discontent.—I

**Published:** 1906-10-20

**Authors:** 


### The Hospital

A Journal of

The Medical Sciences and Hospital Administration.

Vol. XLI.—No. 1,048. SATURDAY, OCTOBER 20, 1906.

### THE PRESENT DISCONTENT.—I.

We have heard from many quarters during recent months that the medical profession is in a melancholy plight, and that there are but slight hopes of its recovery. Its opportunities for private and public service, are, we are told, being diminished on all hands, unqualified competition in various forms is becoming increasingly active, and professional incomes are seriously on the down grade. This, in itself, is a very sorry picture, and the gloom of it is deepened by the inability of the prophets to find a remedy in which they can share a common confidence. Some advise a more strenuous union in the professional ranks, others hurl reproaches against the powers that be, whilst a third group clamour for the strong arm of the law, and from yet another quarter come demands for further parliamentary assistance. In these circumstances it becomes important that members of the profession should view the position with steady gaze, and should cultivate the virtue of clear thinking. Especially is it essential that exaggerated statements should be avoided and that a remedy for existing evils should be sought in the right quarter.

It must be admitted at the outset of the discussion that substantial ground for the present discontent undoubtedly exists. When every allowance is made for the melancholy wail of the pronounced pessimist, and for the strident note of the self-confident reformer, it yet remains true that many sane and sensible practitioners find circumstances increasingly hostile and their professional life decidedly difficult. Hence it is not altogether surprising that professed remedies exercise some attraction.

Among the powers invoked for aid in the present difficulties of the medical profession stand conspicuously the General Medical Council and the High Court of Parliament. The former is the subject of frequent abuse for its supposed indifference to the woes of the profession, and for its unwillingness to conduct an active crusade against extra-professional competition. On the other hand, Parliament hears, or rather is asked to hear, a petition for assistance which is presented with a perseverance truly pathetic. Neither of these lines of movement is by any means a new one, yet so far they have failed to conduct the profession any appreciable distance toward the desired haven. And so far as we are able to judge they are not likely to prove more successful in the future than they have been in the past. Indeed, it seems to us essential that the profession shall cease to cherish the notion that the Medical Council can do much to help the present position even if it were willing to make the attempt, whilst to crave the assistance of Parliament is as useless as to beat the air.

The true position and function of the General Medical Council have recently been admirably defined in an address delivered by the President, Dr. Donald MacAlister. In this is shown how scanty is the authority of the Council, and how the Council exists not to protect the medical profession against competition, but to secure in the public interest the adequate training, due registration, and seemly conduct of qualified medical practitioners. It is neither a medical parliament nor a medical trades union. It can neither produce legislation, nor can it regulate professional practice, and to censure it for failing to do either the one or the other is simply to advertise one's ignorance of its constitution and purpose. That the general body of the profession should be adequately represented on the Council is only right and proper, more especially in view of the penal or disciplinary powers with which it is charged. But no increase in the number or quality of the so-called "direct representatives" can make the Council other than what it is or can enable it to do what by the terms of its constitution it is specifically prevented from undertaking.

But if the General Medical Council is unable to render help, Parliament, it is urged, is all-powerful, and here, then, is to be found the much-desired Jupiter. To Parliament, therefore, a duly drafted Bill is presented containing, *inter alia*, clauses which it is hoped will put down unqualified practice. Upon the construction of this Bill a vast amount of time and ingenuity have been spent, and its terms have been debated and re-debated in that great multitude of meetings provided by the organisation of the British Medical Association. The question has yet to be asked whether, allowing the ability of Parliament, it is possible to command the will of that assembly For ourselves, we gravely doubt that possibility. Legislation which favours, or even appears to favour, a class as distinct from the whole community is at once suspect and arouses distrust as opposed to the instincts and traditions of the British peoples. That Parliament may be persuaded to take any steps that may be necessary to enable the public to distinguish between the qualified and unqualified practioner is readily credible; but that it will in the present state of public opinion attempt to restrict the choice of the public to qualified practitioners only is, we believe, a vain and fond delusion. What the remote future may bring it is impossible to say. But at present we see no reasonable expectation of any such legislation. From this it follows that as the Medical Council is unable, and Parliament is unwilling, to modify the present position it is not to either of these bodies that the medical profession can usefully look for salvation.

